# The Action of Medicines on the System; or, “on the Mode in Which Therapeutic Agents, Introduced into the Stomach, Produce Their Peculiar Effects on the Animal Economy”

**Published:** 1853-07

**Authors:** 


### REVIEWS.

ART. IV.—*The Action of Medicines on the System; or, "on the mode in which therapeutic agents, introduced into the stomach, produce their peculiar effects on the animal economy."* Being the Prize Essay to which the Medical Society of London awarded the Fothergillian gold medal for 1852. By FREDERICK WILLIAM HEADLAND, B. A., M. R. S., etc. Philadelphia: Lindsay & Blakiston, 1853.

The work before us is a decided proof of the revolutionary character of medical science. But a few years ago, many of the high authorities in medical teaching considered that Humoralism, in which they included almost every conceivable form of error in medicine, was forever down; that there was not a remote chance even for one of its multitudinous forms of error to spring into being again, and that the breaking up of that empire of night had paved the way for a glorious state of things in medicine, which might almost be regarded as a millenium. As a converse to all this, it was strongly urged that solidism was but a synonyme for truth; that it contained all vital truth in medicine; that its inculcations had cleared the triumphant progress of medical science from a host of errors that had pinned it to the earth through epochs of time, and that henceforth the "true sun of medical science was high in the ascendant." When Andral began his labors, he was long looked upon with dubiety, and his greatest truths were received with strong suspicions. He was labeled a humoralist, and was shunned as though he were a bale of goods that had been put up in some district of Egypt afflicted with the Oriental Plague, from which noxious pestilence might be caught. But times have changed; humoralism has slowly, but triumphantly worked its success, and its truths are now admitted in almost every quarter. The exclusiveness of solidism died hard, but it died quite effectually.

The author of the work before us is a strong advocate of the humoral school, and his work is a welcome addition to the literature of therapeutics. His introductory remarks are of an excellent character, and they fully reveal the comprehensive manner in which he has studied his subject.

The second chapter is devoted to "some of the more important classifications of medicines, and opinions of authors respecting their action"

Chapter third is "on the general modes of action of therapeutic agents introduced into the stomach; treated of in ten propositions," which we quote, in order to give some idea of the character of the book.

Prop. 1. That the great majority of medicines must obtain entry into the blood or internal fluids of the body, before their action can be manifested

Prop. 2. That the great majority of medicines are capable of solution in the gastric or intestinal secretions, and pass without material change, by a process of absorption, through the coats of the stomach and intestines, to enter the capillaries of the Portal system of veins.

Prop. 3. That those medicines which are completely insoluble in water, and in the gastric and intestinal juices, cannot gain entrance into the circulation.

Prop. 4 That some remedial agents act locally on the mucous surface, either before absorption or without being absorbed at all. That they are chiefly as follow:

*a.* Irritant Emetics.
*b.* Stomach Anæsthetics.
*c.* Irritant Cathartics.

Prop. 5. That medicine, when in the blood, must permeate the mass of the circulation, so far as may be required to reach the parts on which it tends to act.

That there are two possible exceptions to this rule :—

*a.* The production of sensation or pain at a distant point.

*b.* The production of muscular contraction at a distant point.

Prop. 6. That while in the blood the medicine may undergo changes, which in some cases may, in others may not, affect its influence. That these changes may be—

*a.* Of Combination.

*b.* Of Reconstruction.

*c.* Of Decomposition.

Prop. 7. That a first class of medicines, called Hæmatics, act while in the blood, which they influence. That their action is permanent.

1. That of these some, called Restoratives, act by supplying, or causing to be supplied, a material wanting; and may remain in the blood.
2. That others, called Catalytics, act so as to counteract a morbid material or process ; and must pass out of the body.

Prop. 8 That a second class of medicines, called Neurotics, act by passing from the blood to the nerves or nerve-centres, which they influence. That they are transitory in action.

1. That of these some, called Stimulants, act so as to exalt nervous force, in general or in particular.
2. That others, called Narcotics, act so as first to exalt nervous force, and then to depress it ; and have also a special influence on the intellectual part of the brain.
3. That others again, called Sedatives, act so as to depress nervous force, in general or in particular

Prop. 9. That a third class of medicines, called Astringents, act by passing from the blood to muscular fibre, which they excite to contraction.

Prop. 10. That a fourth class of medicines, called Eliminatives, act by passing out the blood through the glands, which they excite to the performance of their functions.

The ten propositions are handled with a high degree of skill, and very fully vindicate the soundness of the judgment of the London Medical Society in presenting this work to the study of the profession. The following is he "classification of medicines which act after entering the blood, according to their supposed modes of operation," adopted by the author :—

CLASS I. HÆMATICA—

*Div.* 1. *Restaurantia*—Ordo 1. Alimenta. Ordo 2. Acida. Ordo 3. Alkalia. Ordo 4. Tonica. Ordo 5 Chalybeata. Ordo 6. Solventia.

*Div.* 2. *Catalytica*—Ordo 1. Antiphlogistica. Ordo 2. Antisyphilitica. Ordo 3. Antiscrofulosa. Ordo 4. Antiarthritica. Ordo 5 Antiscorbutica. Ordo 6. Antiperiodica. Ordo 7. Anticonvulsiva. Ordo 8. Antisquamosa.

CLASS II. NEUROTICA—

*Div.* 1. *Stimulantia*—Ordo 1. Stimulantia Generalia. Ordo 2. Stimulantia Specifica.

*Div.* 2. *Narcotica*—Ordo 1. Inebriantia. Ordo 2. Somnifera. Ordo 3 Deliriantia.

*Div.* 3. *Sedantia*—Ordo 1. Sedantia Generalia. Ordo 2. Sedantia Specifica.

CLASS III. ASTRINGENTIA—

Ordo 1. Astringentia Mineralia. Ordo 2. Astringentia Vegetabilia.

CLASS IV. ELIMINANTIA—

Ordo 1 Sialagoga. Ordo 2 Expectorantia. Ordo 3. Cathartica. Ordo 4. Cholagoga. Ordo 5. Diaphoretica. Ordo 6. Diuretica.

The introductory remarks of the author contain a fair synopsis of the views of the work, on each of the ten propositions we have quoted, and in order to give our readers some insight into the character of Dr. Headland's labors, we quote this synopsis in full.

"Let us consider, as it were, the history of a remedy from the beginning to the end of its course. It is already "introduced into the stomach"—we must commence with it there. Now it does not remain there. It cannot act from the surface of the stomach through the medium of the nervous system.

In the *First Proposition* it is affirmed that it must obtain entry into the fluids of the body—pass, that is, from the intestinal canal into the system at large—before its action can begin. There are four proofs of this. It is shown that when introduced at another part of the body a medicine acts in the same way as when placed in the stomach. It is found by direct experiment that a poison will not act through the medium of nerves only, but that its passage in the blood is required. Thirdly, the course of the circulation is quick enough for the most rapid poison or medicine to pass quite round the body from the veins of the stomach before it begins to operate. The last and most conclusive argument to show that medicines pass out of the stomach into the system, is that they have actually been detected by chemists, not only in the blood, but in the secretions formed from the blood. Remedies, then, pass from the stomach into the blood and fluids. How do they do so?

In the *Second Proposition* it is laid down that all those which are soluble in water, or in the secretions of the stomach or intestines, pass through the coats of these organs into the interior of the capillary veins which surround them. It has already been shown that most medicines pass through in some way; we shall now have to learn how they pass, and what special arrangements are made for the passage of substances differing in nature. By the physical process of absorption a liquid may pass through the animal membranes, from the interior of the stomach or intestine to the interior of the small vein which lies close outside it. In examining the laws by which this process is conducted, we shall find that all the requirements are present in these parts, provided only that the substance to be absorbed shall be first in some way dissolved, and reduced to the liquid state. In the stomach there is, in contact with the substance just introduced, a thin watery secretion containing acid and a matter called pepsin; this is the gastric juice. A large number of medicines are soluble in water. They are dissolved in this fluid. Some others are soluble in dilute acid. These too are dissolved here. Albumen, and matters like it, are reduced to solution by the aid of the pepsin, which is the principle of digestion. But there are some few mineral bodies, and many vegetable substances, as fats and resins, which cannot be thus dissolved by the juice of the stomach. They are soluble, more or less, in a weak alkaline fluid; and such a fluid is the bile, which is poured out into the first portion of the intestine. They too are reduced to solution and absorbed. In this manner it is shown that a very great majority of remedial agents are capable of being reduced to solution, of being absorbed without material change, and of passing thus into the circulation.* Very few are quite insoluble; but some that are dissolved with difficulty may be left partly undissolved in the intestinal canal. What becomes of these?

It is asserted in the *Third Proposition* that substances which are thus insoluble cannot pass into the circulation. Arguing from a physical law, we should say at once that it was impossible; but the matter cannot be so lightly dismissed, for a foreign professor has lately asserted that insoluble matters may and do pass into the circulttion. I have made experiments to satisfy myself on the point, and have come to the contrary conclusion.

In the *Fourth Proposition* it is stated that some few substances may act locally, by irritation or otherwise, on the mucous surface of the stomach or intestines. These are not many; they act without being absorbed; and they do not extend into the system at large. In some few cases, these local actions may be succeeded by changes in distant parts, on the principle of *Revulsion*.

Having just shown how medicinal substances are absorbed, we have now to suppose that they are in the blood.

It is next maintained, in the *Fifth Proposition*, that the medicine, being in the blood, must permeate the mass ot the circulation as far as to reach the part on which it tends to act. This it can easily do. The circulating blood will conduct it any where, in a very short time. Supposing a medicine has to act on the liver, or on the brain, or on the kidney, it does not influence these organs

---

* There is no doubt that the small veins which ramify outside the coats of the stomach and intestines are capable of taking up any matters in a state of proper solution, even fats when dissolved in alkali. But are medicines ever taken up by lacteal absorbents? Probably seldom or never; for it seems that these vessels are only engaged after a full meal, and subsequent to the regular formation of chyle. They do not exist in the coat of the stomach, but commence in the small intestine at some distance from the pylorus.

at a distance, but it passes directly to them in the blood, and then its operation is manifested. This may be called the rule of *local access.* Its proof depends on two things: on the impossibility of the medicinal influence reaching the part in any other way, as shown in the first proposition; and on the fact of medicinal agents having been actually detected in many cases in the very organs over which they exert a special influence. But are there any exceptions to this? Can a medicine ever produce an effect without actually reaching the part? It seems that there may be two exceptions. In some cases an impression of *pain* may be transmitted along a nerve from one part to another; and in some other few instances a muscle, when caused to contract by the influence of a medicine, may cause other muscles near it to contract by sympathy.

Before we inquire into the remedial action of the medicine in the blood, we must consider whether that fluid may not first alter it in some way, so as to hinder or affect its operation. To a certain extent this is possible. In the *Sixth Proposition* it is asserted that while in the blood the medicine may undergo change, which change may or may not affect its influence. It will have to be shown that this change may be one of *combination*, as of an acid with an alkali; of *reconstruction*, when the elements of a body are arranged in a different way, without a material change in its medical properties, as when benzoic is changed into hippuric acid; or of *decomposition*, when a substance is altogether altered or destroyed, as when the vegetable acids are oxidized into carbonic acid.

Having considered these preliminary matters, we shall arrive at the main point. The medicines are now in the blood. We must consider what becomes of them; what they do next; where they go next; and how they operate in the cure of diseases. I have made a classification in which medicines are divided according to my views of their mode of operation. The classes and their subdivisions will serve for references in illustration of what I have to say. For it is not possible to speak of the general operation of medicines without adducing particular instances; nor will time and space always allow me, in doing so, to refer to individual medicines.

There are four great groups of medicines, the action of each of which is well marked and distinct. The first class acts in the blood; and as a large number of diseases depends on a fault in that fluid, we may by their means be enabled to remedy that fault. They are the most important of all medicines. They are called Hæmatics, or blood-medicines. They are used chiefly in chronic and constitutional disorders. But a second class of remedies are temporary in their action. They influence the nervous system, exciting it, depressing it, or otherwise altering its tone. They are chiefly useful in the temporary emergencies of acute disorders. They can seldom effect a permanent cure, unless when the contingency in which they are administered is also of a temporary nature. They are called Neurotics, or nerve-medicines. A third set of medicines, less extensive and less important than the others, acts upon muscular fibre, which is caused by them to contract. Involuntary muscular fibre exists in the coats of small bloodvessels, and in the ducts of glands. Thus Astringents, as these agents are called, are able, by contracting muscular fibre, and thus diminishing the calibre of these canals, to arrest hemorrhage in one case (when a small vessel is ruptured,) and to prevent the outpouring of a secretion in another case.

The fourth class is of considerable importance. Some medicines have the power of increasing the secretions which are formed from the blood by various glands at different parts of the body. By their aid we may be enabled to eliminate from the blood a morbid material through the glands; or we may do great good by restoring a secretion when unnaturally suppressed. They are called Eliminatives. Like Hæmatics, their influence is more or less permanent. That of Neurotics and Astringents, particularly the former, is transient.

The general mode of action of these four classes of therapeutic agents is laid down in the four remaining propositions, about as far as it seems to me to be capable of a positive definition. Each proposition concerns one of these classes of medicines. All I can do now is to recapitulate the chief affirmations made; as to give any idea of their proof would require me to enter into a number of details which had better be postponed to the third chapter.

In the *Seventh Proposition* it is stated of Hæmatic medicines that they act while in the blood, over which fluid they exert an influence; and that their effect, whatever it be, is of a more or less permanent character. A line of distinction is drawn between two divisions of this class of blood-medicines. Some of them are natural to the blood; they resemble or coincide with certain substances that exist in that fluid; so that, having entered it, they may remain there, and are not necessarily excreted again. These are useful when the blood is wanting in one or more of its natural constituents. This want causes a disease, and may be supplied by the medicine which in this way tends to cure the disease. Medicines of this division are called Restoritives; for they *restore* what is wanting.

Some other blood-medicines, although they enter the blood, are not natural constituents of the vital fluid, and cannot remain there, for they are noxious and foreign to it. They must sooner or later be excreted from it by the glands. They are of use when disease depends on the presence and working in the blood of some morbid material or agency, which material or action they tend to counteract or destroy. They may be called *vital antidotes*; not strictly *specifics*, for they are not always efficacious, on account of variations in the animal poisons, or from the casual operation of disturbing causes. They are applicable in those many disorders which depend, not on the absence of a natural substance, but on the presence of an unnatural agent in the blood. These medicines are called Catalytics, from a Greek word which signifies *to break up* or *to destroy*. Having performed this, their function, they then pass out of the blood.

All this requires to be proved.

In the *Eigth Proposition* it is stated of Neurotics, or nerve-medicines, that they act by passing out of the blood to the nerves, which they influence. This is only to insist on the rule of *local access*, already laid down in Prop. V. It is further affirmed that they are transitory in action. They appear to affect molecular changes in nerve-fibre, similar to those by which the phenomena of the senses are produced, and which are by nature transitory in their results. And yet they may be very powerful, even so as to extinguish vital force. Thus, short and unenduring as is the operation of these agents, it may last long enough to cause death, and so a temporary influence produce a permanent result. There are three divisions of Neurotics. The first set are of use when there is a dangerous deficiency of vital action. These are stimulants. They exalt nervous force, either of the whole nervous system, or only of a part of it. They vary very much in power. A second set, called Narcotics, first exalt nervous force, and then depress it. They have thus a double action; but they have also a peculiar influence over the functions of the brain, which is different from any possessed by other nerve medicines. They control the intellectual part of the brain, as distinguished from its organic function; the powers of *mind* more than those of *life*. Some Narcotics tend to produce inebriation; others, sleep; others, again, delirium. In the third place some Neurotics tend simply and primarily to depress nervous force. They may act on the whole nervous system, or on a part of it only. They are often very powerful; and they are of use when, from any cause, some part of the nervous system is over-excited. They are called Sedatives. Like other Neurotics, they are used in medicine as temporary agents in temporary emergencies. If a permanent action be required, the remedy must be constantly administered, so that the effect may be kept up by continual repetition.

In the *Ninth Proposition* it is affirmed of Astringent medicines that they act by passing out of the blood to muscular fibre, which by their contact they excite to contraction. They do not so much influence the voluntary fibre of the muscles, which is under the direct control of the nervous system; but they chiefly manifest their action on the involuntary or unstriped muscular fibre, which is not directly controlled by the brain and nerve-centres, and for this reason more under the operation of external or irritating agents. Meeting this in the coats of the capillary vessels and of the ducts of glands, they are enabled to act as styptics, and as checkers of secretion. The action of Astringents appears to depend on a chemical cause; for we find that all of them possess the power of coagulating albumen.

The *Tenth Proposition* treats of Eliminatives. It is not said simply that these increase the secretions of a gland; or that they stimulate the glands while passing by them in the blood. But it is laid down as a rule that they act by themselves passing out of the blood through the glands, and that while so doing they excite them to the performance of their natural function. They are substances which are unnatural to the blood, and must therefore pass out of it. In so doing they tend to pass by some glands rather than by others: in these secretions they may be detected chemically; and it is on these glands they have an especial influence. Their uses in treatment are various and manifold."

